# Dr. Simon A. Freeman

**Published:** 1906-02

**Authors:** 


### OBITUARY.

Dr. Simon A. Freeman, formerly of Everett, Mass., more recently of California, lately died at his home in that state. He was a hospital steward during the civil war, and served as acting assistant surgeon United States Army from 1870 to 1879. He graduated at the Medical Department of the University of Buffalo in 1869.

